# Preparation, Characterization, and Performance Analysis of S-Doped Bi_2_MoO_6_ Nanosheets

**DOI:** 10.3390/nano9091341

**Published:** 2019-09-19

**Authors:** Ruiqi Wang, Duanyang Li, Hailong Wang, Chenglun Liu, Longjun Xu

**Affiliations:** 1 State Key Laboratory of Coal Mine Disaster Dynamics and Control, Chongqing University, Chongqing 400044, China; wrq666@cqu.edu.cn (R.W.); cqwhl@cqu.edu.cn (H.W.); 2 College of Chemistry and Chemical Engineering, Chongqing University, Chongqing 401331, China; 20161813101@cqu.edu.cn

**Keywords:** S-doped Bi_2_MoO_6_, Rhodamine B, photodegradation rate, characterization, performance

## Abstract

S-doped Bi_2_MoO_6_ nanosheets were successfully synthesized by a simple hydrothermal method. The as-prepared samples were characterized by X-ray diffraction (XRD), scanning electron microscope (SEM), transmission electron microscopy (TEM), N_2_ adsorption–desorption isotherms, Raman spectroscopy, Fourier transform infrared spectroscopy (FT-IR), elemental mapping spectroscopy, photoluminescence spectra (PL), X-ray photoelectron spectroscopy (XPS), and UV-visible diffused reflectance spectra (UV-vis DRS). The photo-electrochemical performance of the samples was investigated via an electrochemical workstation. The S-doped Bi_2_MoO_6_ nanosheets exhibited enhanced photocatalytic activity under visible light irradiation. The photo-degradation rate of Rhodamine B (RhB) by S-doped Bi_2_MoO_6_ (1 wt%) reached 97% after 60 min, which was higher than that of the pure Bi_2_MoO_6_ and other S-doped products. The degradation rate of the recovered S-doped Bi_2_MoO_6_ (1 wt%) was still nearly 90% in the third cycle, indicating an excellent stability of the catalyst. The radical-capture experiments confirmed that superoxide radicals (·O^2−^) and holes (h^+^) were the main active substances in the photocatalytic degradation of RhB by S-doped Bi_2_MoO_6_.

## 1. Introduction

With the rapid development of textile, metallurgical, chemical, and other industries, the discharge of industrial wastewater such as organic wastewater and dye wastewater has gradually increased. Increasingly serious water pollution poses a huge potential threat to people’s health and lives. In 1972, Fujishima et al. [1] found for the first time that under ultraviolet excitation, water could be photocatalyzed by single-crystal TiO_2_ electrodes to produce clean products: H_2_ and O_2_. In 1976, TiO_2_ was successfully applied to photocatalytic degradation of polychlorinated biphenyls. Since then, more semiconductor photocatalysts have been found to be capable of photocatalytic degradation of organic macromolecular contaminants in wastewater [2,3]. Therefore, photocatalytic degradation has become the most environmentally friendly, energy-saving, and efficient water pollution treatment method. In view of the fact that the traditional photocatalysts (such as TiO_2_) have large band gap energy and low response to visible light, their application is greatly limited. Among the semiconducting photocatalysts, bismuth molybdate (Bi_2_MoO_6_) as a ternary oxide compound of Aurivillius phase becomes one of the promising materials. This is because it has a unique layered structure sandwiched between the perovskite octahedral (MoO_4_)^2−^ sheets and bismuth oxide layers of (Bi_2_O_2_)^2+^ [4,5,6]. Its dielectric property, ion conductivity, and catalytic performance have obvious advantages in bismuth-based semiconductors [7,8]. Nevertheless, the light absorption property of the pure Bi_2_MoO_6_ primarily appears in the ultraviolet light region, which is only a small part of the solar spectra. Meanwhile, it presents a high recombination rate of electron-hole pairs in the process of photocatalytic reaction [9]. Therefore, researchers have improved the performance of Bi_2_MoO_6_ by means of morphology controlling, semiconductor compounding, and doping modification [10]. Among these measures, doping has proven to be an effective method to ameliorate the surface properties of photocatalysts and enhance photocatalytic performance.

It was reported that carbon-doped Bi_2_MoO_6_ exhibited significantly enhanced and stable photocatalytic properties compared with Bi_2_MoO_6_ [11], which carbon replaced the O^2−^ anion in the lattice of Bi_2_MoO_6_, resulting in lattice expansion and grain diameter reduction, enhancement of specific surface area. Wang et al. [12] prepared Graphene-Bi_2_MoO_6_ (G-Bi_2_MoO_6_) hybrid photocatalysts by a simple one-step process, and an increase in photocatalytic activity was observed for G-Bi_2_MoO_6_ hybrids compared with pure Bi_2_MoO_6_ under visible light. Xing [13] reported the photocatalytic activity of 0.5% Pd–3C/BMO was robustly enhanced about 5-fold for Rhodamine B (RhB) degradation within 40 min under UV + visible light irradiation and 29-fold for O-phenylphenol (OPP) degradation within 120 min under visible light irradiation in comparison with pristine Bi_2_MoO_6_, respectively. Wang [14] prepared a B-doped Bi_2_MoO_6_ photocatalyst with hydrothermal method by using HBO_3_ as a dopant source. It was found that B-doping increases the amount of Bi^5+^ and oxygen vacancies, so that the visible light absorption of catalyst is stronger, and the band gap energy is lower, which significantly improves the photocatalytic activity of Bi_2_MoO_6_. Chen [15] successfully synthesized sulfur-doped copper-cobalt bimetal oxide by coprecipitation method, which significantly improved the catalytic performance and stability of the catalyst. Wang [16] fabricated Bi_2_MoO_6_ surface co-doped with Ni^2+^ and Ti^4+^ ions through an incipient-wetness impregnation technology and calcination method, with the results suggesting Ni^2+^ and Ti^4+^ co-doping increases visible-light absorption by Bi_2_MoO_6_ and promotes the separation of photogenerated charge carriers. Density functional theory calculations and systematical characterization results revealed that Bi self-doping could not only promote the separation and transfer of photogenerated electron-hole pairs of Bi_2_MoO_6_ but also alter the position of valence and conduction band without changing its preferential crystal orientations, morphology, visible light absorption, as well as band gap energy [17]. Zhang et al. [18] synthesized pure and various contents of Ce^3+^ doped Bi_2_MoO_6_ nanostructures by a facile hydrothermal method. The 0.5% Ce^3+^ doped Bi_2_MoO_6_ exhibits the best photocatalytic activity of 96.6% within 20 min for RhB removal.

It can be inferred from the above studies that sulfur doping may improve the photocatalytic property of Bi_2_MoO_6_. However, the photocatalytic performance of S-doped Bi_2_MoO_6_ nanoparticles has been scarcely reported so far. In this work, a hydrothermal method was used to synthesis the S-doped Bi_2_MoO_6_ visible light catalysts. The crystal physical structure, microscopic morphology, elemental valence state, and optical performance were studied using X-ray diffraction (XRD), scanning electron microscope (SEM), transmission electron microscopy (TEM), Fourier transform infrared spectroscopy (FT-IR), X-ray photoelectron spectroscopy (XPS), photoluminescence spectra (PL), N_2_ adsorption–desorption, elemental mapping, Raman and diffused reflectance spectra (DRS) analysis. The photo-degradation rate of RhB under visible light was used to appraise the photocatalytic performance of S-doped Bi_2_MoO_6_ samples.

## 2. Experiments 

### 2.1. Preparation of Photocatalyst

S-doped Bi_2_MoO_6_ nanosheets were prepared by a simple hydrothermal method. In this experiment, 2.5 mmol of Bi(NO_3_)_3_·5H_2_O were ultrasonically dissolved in 10 mL of ethylene glycol to obtain solution A. Simultaneously, 0.5 mmol of (NH_4_)_6_Mo_7_O_24_·4H_2_O and 0.2 g of polyvinylpyrrolidone (PVP) were ultrasonically dissolved in 20 mL of deionized water to get mixture B. The solution A was slowly added dropwise into the mixture B under continuous magnetic stirring, after that amorphous white precipitate formed immediately. After being vigorously stirred for 15 min, the the pH value of the mixed solution was adjusted to 9 with sodium hydroxide solution, and pale yellow precipitate was obtained. Afterwards, the thiourea of different mass ratios were added into the precursor solution, and the obtained mixed solution was all transferred into a 50 mL polytetrafluoroethylene-lined stainless steel autoclave, then kept it at 120 °C for 4 h and taken out. After the autoclave naturally cooled to room temperature, the obtained sample was separated, washed several times using deionized water and absolute ethanol, and dried in an oven at 60 °C for 12 h. Then, the S-doped Bi_2_MoO_6_ sample was obtained. For reference, the pure Bi_2_MoO_6_ was produced by a similar method.

### 2.2. Characterization

The crystal structure and composition of the photocatalyst materials were identified by XRD (XRD600, Shimadzu, Kyoto, Japan). The molecular structure of the samples was characterized through FT-IR (5DXFT-IR, Lake Shore, Columbus, OH, USA). The elemental valence states were analyzed via XPS (ESCALAB 250Xi, Thermo Fisher Scientific, Shanghai, China). The microstructure, morphology and lattice sizes of the materials were observed using SEM (QUANTA250, FEI, Hillsboro, OR, USA), TEM (JEM-2100F, JEOL, Akishima City, Tokyo, Japan) and high resolution TEM. The N_2_ adsorption–desorption isotherm was obtained at a liquid nitrogen temperature of –195.8 °C using an ASAP 2020 Plus HD88 (Micromeritics, Norcross, GA, USA) gas adsorption and porosity analyzer to test the specific surface area and pore size distribution of the sample. The photoluminescence properties were tested via the fluorescence spectrophotometer (Cary eclipse, NYSE: A, Palo Alto, Santa Clara, CA, USA) and the excitation wavelength was 300 nm. The molecular structure was analyzed qualitatively using the Raman spectrometer (LabRAM HR Evolution, France HORIBA Jobin Yvon S.A.S, Paris, France). The band gap of the catalysts was tested by the UV-visible diffuse reflection spectra (UV-Vis DRS, TU-1901, Beijing Puxi, Beijing, China) over the wavelength range between 200 and 800 nm. 

### 2.3. Determination of Photocatalytic Property

The photo-degradation rate of RhB solution under visible light irradiation was used to appraise the photocatalytic properties of samples. In this photocatalytic experiment, the dye wastewater was simulated with RhB solution, and a 300 W Xenon lamp (CEL-HXF300, Zhongjiaojinyuan, Beijing, China) was used as the light source. During the reaction, 100 mg of photocatalysts were weighed and added into 100 mL of RhB solution (10 mg/L), then the mixed solution was placed in the dark and stirred for half an hour to realize the absorption–desorption equilibrium. After the Xenon lamp was turned on, the vertical height between the liquid level and the light source was kept at 20 cm while magnetic stirring and the cut-off filter used was 420 nm. Afterwards, 3 mL of the mixed solution was removed at intervals of 15 min, and then centrifuged at 3900 r/min for 2 min. The supernatant after centrifuging was measured for absorbance (λ _max_ = 554 nm) to calculate the degradation rate of RhB.

### 2.4. Determination of Photoelectrochemical Property

Electrochemical impedance spectroscopy and photocurrent density were measured via an electrochemical workstation (CHI-660E, Chenhua, Shanghai, China). A 300 W Xenon lamp was used to illuminate and the cut-off filter used was 420 nm. The three-electrode system was established for testing, which used Ag/AgCl, platinum sheets and the photocatalyst coated F-doped tin oxides (FTO) film electrode as reference, counter, and working electrodes, respectively. Simultaneously, the electrolyte was composed of 0.1 mol/L Na_2_SO_4_ solution. The preparation details of FTO thin film electrode was as follows. The FTO glass was ultrasonically cleaned with absolute ethanol for 10 min. After drying, the conductive surface was measured with a multimeter. Then, 20 mg of photocatalyst was weighed and dispersed in 10 μL (0.5%) of nafion solution, 200 μL of absolute ethanol, and 200 μL of distilled water by sonicating for 30 min. The 100 μL solution was pipetted on the FTO conductive surface and spread evenly with a spin coater. The working area was 1 × 1 cm. Then, the solution was dried and prepared for subsequent testing.

## 3. Results and Discussion 

### 3.1. Photocatalytic Property

**Figure 1:**
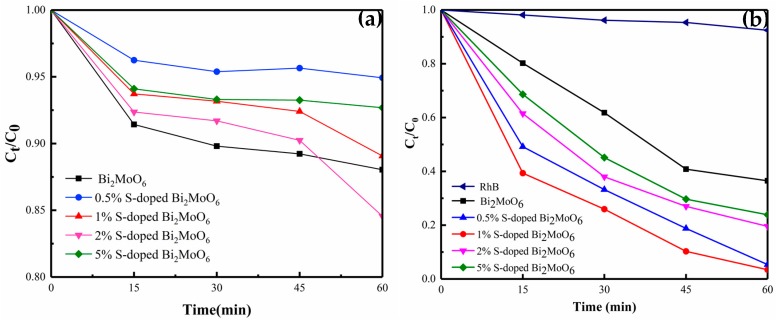
(**a**) The adsorption curves of pure Bi_2_MoO6 and Bi_2_MoO_6_ with different S doping amounts in the dark, (**b**) the degradation rate of pure Bi_2_MoO_6_ and Bi_2_MoO_6_ with different S doping amounts under visible light

Figure 1a shows the adsorption curves of the prepared samples under dark condition. It can be seen from the image that the dark reaction adsorption rates of the samples after S doping were slightly decreased, but all were kept within 15% after 60 min. The photocatalytic properties of Bi_2_MoO_6_ with different contents of S doping were assessed by the degradation rate of RhB under visible light. It can be observed from Figure 1b that the photo-degradation efficiency reached up to 64% by the pure Bi_2_MoO_6_ after 60 min light, and the degradation rate of S-doped Bi_2_MoO_6_ with the sulfur contents increased (0.5%, 1%, 2%, 5%) showed a trend of increasing first and then decreasing (95%, 97%, 80%, 77%). The results show that S doping is beneficial to improve the photocatalytic activity. Among them, 1% S-doped Bi_2_MoO_6_ exhibited the best photocatalytic performance and its degradation rate was 97% after 60 min visible light irradiation, which was higher than that of the pure Bi_2_MoO_6_ (64%) and other S-doped products. 

### 3.2. Crystal Structure and Composition 

**Figure 2:**
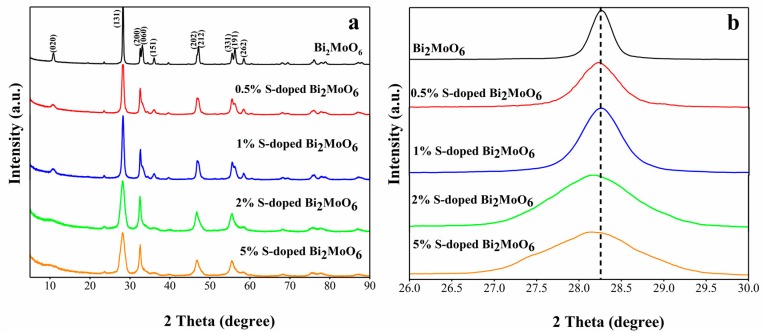
X-ray diffraction (XRD) patterns of samples: (**a**) S-doped Bi_2_MoO_6_, (**b**) local magnification of (131) crystal lane.

The microscopic crystal composition and structure of the pure Bi_2_MoO_6_ and S-doped Bi_2_MoO_6_ samples were identified via X-ray diffraction. As we can see from Figure 2a, all characteristic peaks of S-doped Bi_2_MoO_6_ can be corresponded to the pure Bi_2_MoO_6_ phase with Pca21 space group shown in the standard card (JCPDS No.72-1524), and the lattice parameters are a = 5.506 Å, b = 16.226 Å, c = 5.487 Å. The main characteristic diffraction peaks of S-doped Bi_2_MoO_6_ at 2θ = 27.98°, 32.30°, 46.60°, 55.34° matched with the (020), (131), (002), (202) crystal planes, respectively. The two adjacent bimodal (200) and (060), (202) and (212), (331) and (191) were respectively transformed into a sharper single peak after S doping. There were not any characteristic peaks in the XRD pattern of S-doped Bi_2_MoO_6_ of other impurities such as Bi_2_S_3_, MoS_2_, etc. It was sufficient to show that S^2-^ replaced O^2−^ in Bi_2_MoO_6_ after S doping, thereby expanded the crystal lattice. This was beneficial to improve photocatalytic performance [19,20].

It can be seen from the partial enlarged view of the crystal plane in Figure 2b that the (131) plane was overall shifted toward a smaller angle. In addition, interestingly, the peak intensities of S-doped Bi_2_MoO_6_ samples first increased and then decreased with an increase of S doping content, while 1% S-doped Bi_2_MoO_6_ exhibited the strongest peak intensities. It indicated that 1% S-doped Bi_2_MoO_6_ sample has the best crystallinity among the as-prepared samples. This conclusion was consistent with the above results that 1% S-doped Bi_2_MoO_6_ has the best photocatalytic properties.

In order to study the changes in the crystalline phase due to the sulfur doping, the crystallite size of the pure Bi_2_MoO_6_ and S-doped Bi_2_MoO_6_ samples were calculated by the Scherrer equation:

(1) $$D=\frac{K\lambda }{B\cos\theta },$$

where *D* is the particle diameter (nm), *K* is the constant (0.89), *λ* is the X-ray wavelength (0.15406 nm), *B* is the half-maximum line width, and *θ* is diffraction angle. We can see from Table 1 that the crystallite sizes of samples decreased from 28.2 to 9.2 nm after S doping. The reason for the decrease may be that the S doping can inhibit the growth of the crystal, thereby decreased the crystal volume. Thus, it can be believed that the photocatalytic activity will be improved by S doping [21]. 

### 3.3. Optical Property

**Figure 3:**
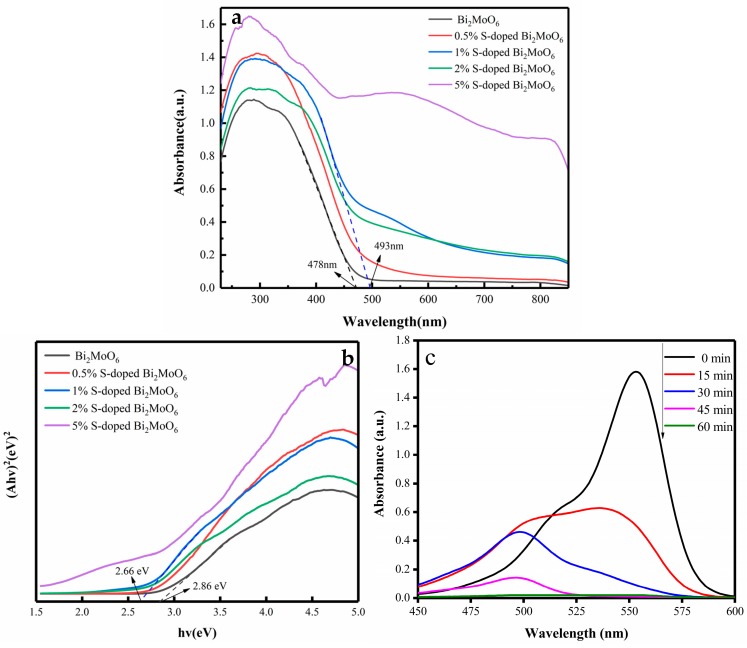
(**a**) UV-visible diffuse reflection spectra of the pure Bi_2_MoO_6_ and Bi_2_MoO_6_ with different S doping amounts, (**b**) band gap width of the pure Bi_2_MoO_6_ and Bi_2_MoO_6_ with different S doping amounts, (**c**) UV-visible absorption spectrum of 1% S-doped Bi_2_MoO_6_ sample.

The optical performance of the as-prepared samples was evaluated via UV-vis DRS. It can be observed from Figure 3a that all the samples exhibited strong absorption in UV and visible light regions. The absorption edge of Bi_2_MoO_6_ sample was located at about 478 nm. After S doping, the light absorption edge of the photocatalysts showed a significant red shift. Among them, the absorption edge of 1% S-doped Bi_2_MoO_6_ was located at 493 nm. The red shift was attributed to the charge-transfer transition between the electrons of the doped S^2−^ and the Bi_2_MoO_6_ conduction band. The phenomena indicated that the absorption property became better after S doping. Generally, the better absorption properties are used to produce more photogenerated carriers, which is more beneficial to the photocatalytic reaction [22,23].

The band gap energies of these photocatalysts are critical for the excitation and transition of electron and hole and can be calculated by the equation as follows:

(2) ahν=A(hν−Eg)n2

where *α* is the absorption coefficient, *ν* is light frequency, *A* is proportional constant, *E*_g_ is the band gap energy, *h* is the Planck’s constant, and *n* is determined by the type of optical transition of a semiconductor. Since Bi_2_MoO_6_ is a direct transition semiconductor, *n* takes one.

Based on Equation (2), the band gap energies of the as-prepared samples can be separately obtained from the plots between (*αhν*)^2^ and photon energy *hν* (Figure 3b). First, a tangent of the curve was made, and then the detailed *E*_g_ value was calculated from the intercept of the tangent. The band gap energy was significantly decreased from 2.86 eV of Bi_2_MoO_6_ to 2.49 eV of 5% S-doped Bi_2_MoO_6_, as shown in Table 1. As we can see the *E*_g_ value of Bi_2_MoO_6_ sample was calculated to be 2.86 eV, which is consistent with the previous studies [24,25]. The *E*_g_ value of the photocatalysts were narrowed after S doping, which means better light absorption capability and it is helpful for the improvement of photocatalytic activity. 

Usually, the photo-degradation rate of RhB solution is used to evaluate the performance of the photocatalyst. Figure 3c shows the UV-visible absorption spectrum of 1% S-doped Bi_2_MoO_6_ when RhB is the target degradant. After being catalytically degraded for 1 h, the absorbance of RhB solution quickly decreased to zero, and the maximum absorption peak gradually moved from 554 to 496 nm. Moreover, the color of RhB solution turned from the purple to colorless and transparent, indicating that the 1% S-doped Bi_2_MoO_6_ sample has an excellent photocatalytic effect.

Through repeated experiments, we concluded that 1% S-doped Bi_2_MoO_6_ has the best photocatalytic activity in the prepared samples. Therefore, the following discussions focus on the characterization and comparison of pure Bi_2_MoO_6_ and 1% S-doped Bi_2_MoO_6_ to study the influencing factors and mechanisms of S doping on the photocatalytic activity of Bi_2_MoO_6_.

**Figure 4:**
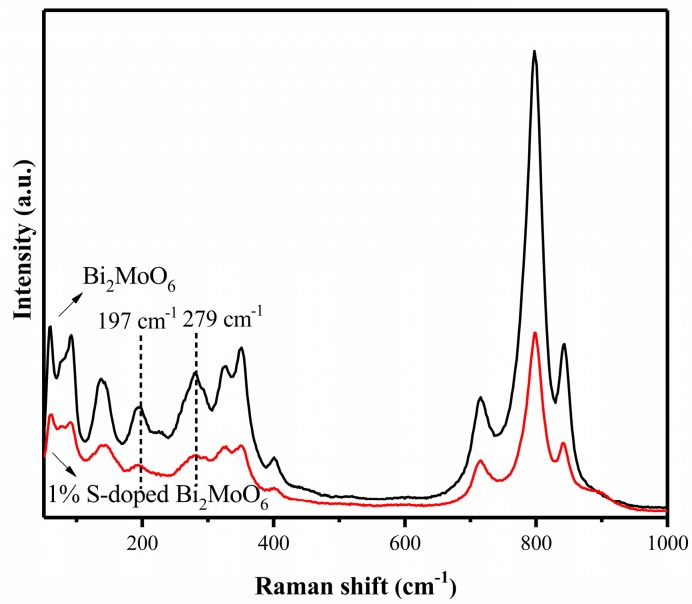
Raman spectra of Bi_2_MoO_6_ and 1% S-doped Bi_2_MoO_6._

Raman spectra of the pure Bi_2_MoO_6_ and 1% S-doped Bi_2_MoO_6_ sample are shown in Figure 4. For Bi_2_MoO_6_, a very strong peak at 797 cm^−1^ was assigned to A_1g_ mode of Mo-O stretching vibration of the MoO_6_ octahedron. The Raman peaks at 715 and 843 cm^−1^ were also designated to A_1g_ mode and corresponded to the orthogonal distortion of the MoO_6_ octahedron. The peaks at 401, 351, 326, and 279 cm^−1^ represented symmetric stretching vibrations of Mo-O bonds, which have wavenumber modes below 205 cm^−1^. Raman peaks at 198, 138, 92, and 60 cm^−1^ could be attributed to the translation of molybdenum and bismuth atoms. After S doping, the Raman peaks of composite photocatalysts were broader and weaker than pure Bi_2_MoO_6_, which indicated that the particle sizes of the S-doped Bi_2_MoO_6_ were decreased. The result was consistent with the calculation value of Scherrer equation. In addition, the Raman peaks of 1% S-doped Bi_2_MoO_6_ at 279 and 198 cm^−1^ was significantly changed compared with pure Bi_2_MoO_6_. It can be further demonstrated that S doping changed the Mo-O bonds in Bi_2_MoO_6_, and O^2−^ in Bi_2_MoO_6_ were successful replaced by S^2−^. The above results were consistent with the literature [26,27].

**Figure 5:**
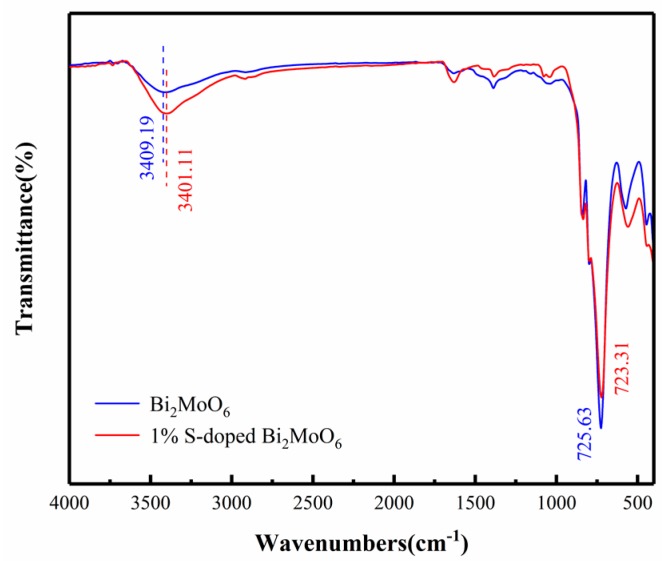
Fourier transform infrared spectroscopy (FT-IR) spectra of Bi_2_MoO_6_ and 1% S-doped Bi_2_MoO_6._

The FT-IR spectra of Bi_2_MoO_6_ and 1% S-doped Bi_2_MoO_6_ are shown in Figure 5. As we can see the characteristic absorption bands at 445 and 579 cm^−1^ respectively corresponded to the Bi-O bond telescopic and deformation vibrations. The absorption bands located at 731, 796, and 841 cm^−1^ were ascribed to the Mo-O stretching vibration in the MoO_6_ octahedron. Those peaks at 3409, 1640, and 1380 cm^−1^ were assigned to the bending and stretching vibration peaks of the O-H bond [28,29]. Compared with the pure Bi_2_MoO_6_, the absorption band of 1% S-doped Bi_2_MoO_6_ caused a slight red-shift, and the strength of the bands at 400–900 cm^−1^ significantly reduced. The above results further confirmed that S^2−^ were successfully doped into the crystal lattice of bismuth molybdate [30,31].

**Figure 6:**
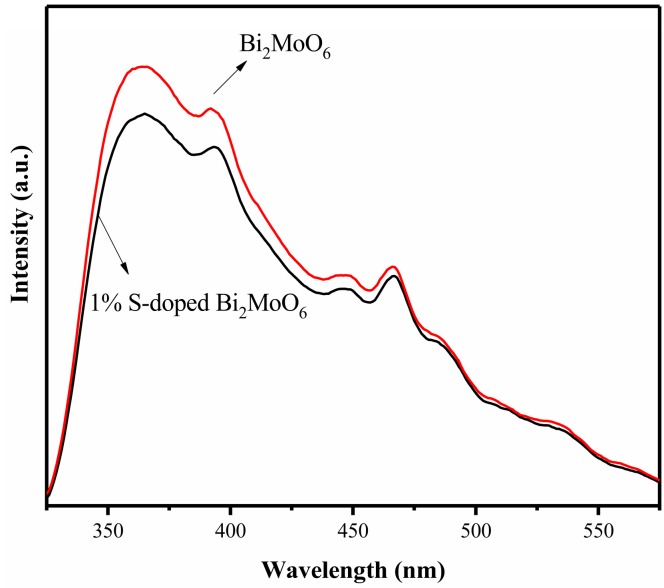
Photoluminescence (PL) spectra of Bi_2_MoO_6_ and 1% S-doped Bi_2_MoO_6._

The PL spectra at an excitation wavelength of 300 nm of pure Bi_2_MoO_6_ and 1% S-doped Bi_2_MoO_6_ are presented in Figure 6. As we can see the PL spectra of the two are similar, but the intensity of fluorescence emission peak of 1% S-doped Bi_2_MoO_6_ is significantly lower than that of Bi_2_MoO_6_. It proved that the combination rate of electrons and holes of 1% S-doped Bi_2_MoO_6_ were lower than Bi_2_MoO_6_ and thus 1% S-doped Bi_2_MoO_6_ showed higher photocatalytic activity. It can be seen that S doping can suppress the recombination of photogenerated electrons and holes [32,33]. The possible reason was that the photogenerated electrons could migrate to these new defect sites which were created by S-doping, thereby resulted in the recombination of photogenerated electrons and caused the holes to reduce.

### 3.4. Morphology and Crystal Analysis

**Figure 7:**
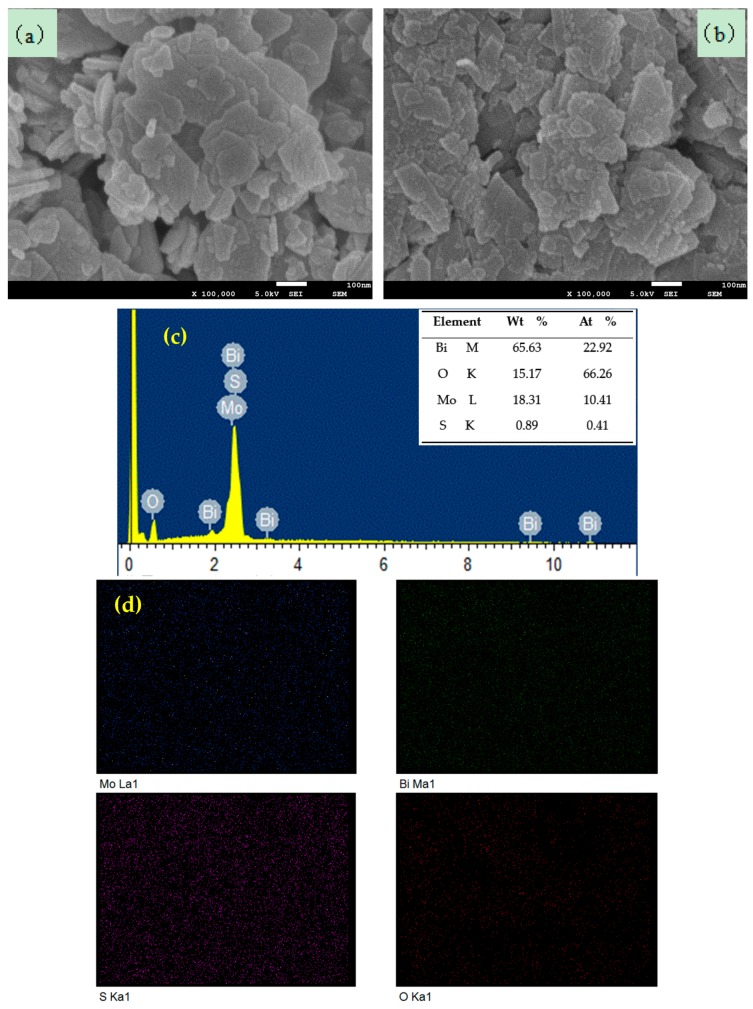
(**a**) Scanning electron microscope (SEM) image of Bi_2_MoO_6_, (**b**) SEM image of 1% S-doped Bi_2_MoO_6_, (**c**) energy dispersive X-ray spectroscopy (EDX) spectrum of 1% S-doped Bi_2_MoO_6_, and (**d**) element mapping images of 1% S-doped Bi_2_MoO_6_.

The microstructural and morphology of the pure Bi_2_MoO_6_ and 1% S-doped Bi_2_MoO_6_ were observed via SEM. It can be seen from Figure 7 that all samples were non-uniform nano-sheet structures. Moreover, there were many differences between S-doped Bi_2_MoO_6_ and pure Bi_2_MoO_6_ in morphology and particle size. As we can see Bi_2_MoO_6_ were comprised of a large number of nanoplates with diameters about 300 nm and thickness about 25 nm (Figure 7a). The 1% S-doped Bi_2_MoO_6_ were comprised of nanoplates with diameters about 150 nm and thickness about 15 nm (Figure 7b). The results showed that the diameters and thickness of 1% S-doped Bi_2_MoO_6_ were smaller than those of the pure Bi_2_MoO_6_. The possible reason was that S^2−^ inhibited crystal growth, which was consistent with the calculation of the crystallite size above.

Figure 7c reveals the energy dispersive X-ray spectroscopy (EDX) of the 1% S-doped Bi_2_MoO_6_ sample. Obvious signals for Bi, O, Mo, and S elements can be observed. The content of S was calculated as 0.89%, which was nearly in keeping with the theoretical value (1%). The corresponding element mapping images presented in Figure 7d displayed the distribution of individual elements Bi, Mo, O, and S in the 1% S-doped Bi_2_MoO_6_ sample, confirming that S were uniformly dispersed in the 1% S-doped Bi_2_MoO_6_.

**Figure 8:**
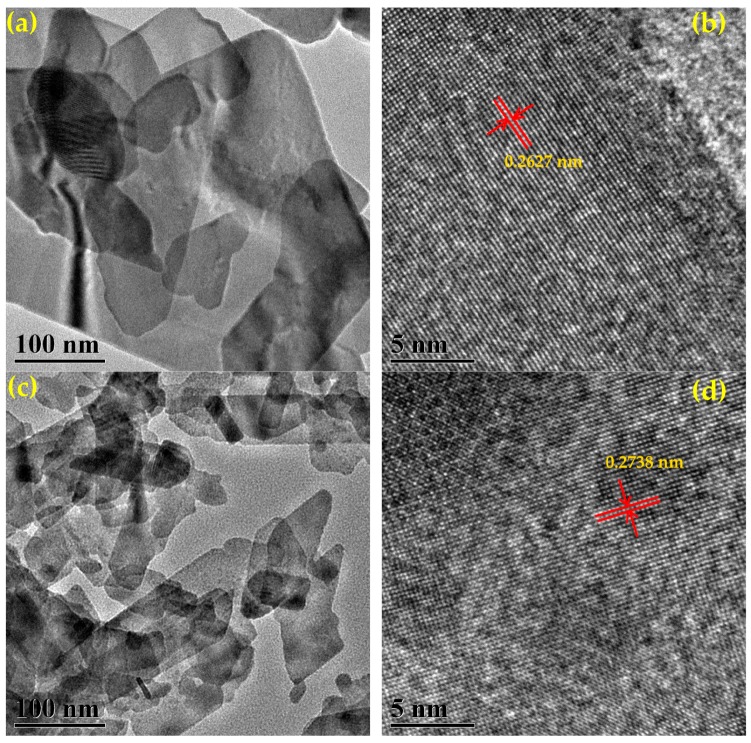
Transmission electron microscopy (TEM) and high resolution TEM (HRTEM) images of Bi_2_MoO_6_ (**a**,**b**) and 1% S-doped Bi_2_MoO_6_ (**c**,**d**).

The TEM and HRTEM images of Bi_2_MoO_6_ and 1% S-doped Bi_2_MoO_6_ are displayed in Figure 8. The TEM images of the samples in Figure 8a,c reveal the nanosheet morphology, which are consistent with the SEM results above. The lattice fringes with the spacing of 0.2627 nm shown in Figure 8b match well with the (060) crystalline plane of Bi_2_MoO_6_. In HRTEM image of Figure 8d, the lattice fringes with the spacing of 0.2738 nm are ascribed to the (200) crystalline plane of Bi_2_MoO_6_. The results show that compared with pure Bi_2_MoO_6_, the morphology of Bi_2_MoO_6_ was not changed significantly after sulfur doping, but the thickness and width of the nanosheets were reduced, and the lattice of Bi_2_MoO_6_ became larger after S doping. This is consistent with the results above.

### 3.5. Chemical Composition and Valence Analysis

**Figure 9:**
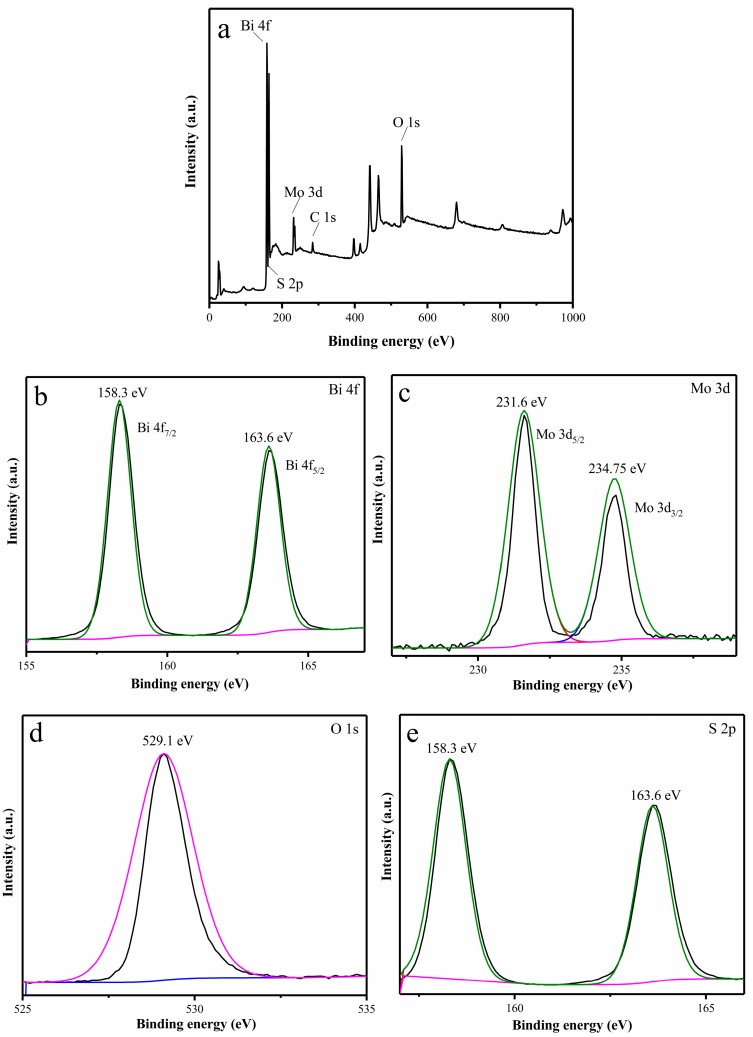
X-ray photoelectron spectra (XPS) of 1% S-doped Bi_2_MoO_6_ sample: (**a**) full spectrum, (**b**) Bi 4f, (**c**) Mo 3d, (**d**) O 1s, and (**e**) S 2p.

The chemical composition and elemental valence state of 1% S-doped Bi_2_MoO_6_ photocatalyst were studied by XPS as shown in Figure 9. It can be observed from the XPS full spectrum that the 1% S-doped Bi_2_MoO_6_ sample was mainly composed of Bi, Mo, O, C, and S, among which a small amount of C might derive from the hydrocarbons of the instrument. From the photoelectron spectroscopy of Bi 4f, we can see that the two peaks located at 158.3 and 163.6 eV were respectively indexed to Bi 4f_7/2_ and Bi 4f_5/2_ with the spin-orbit splitting difference of 5.3 eV, which were attributed to the binding energy of Bi^3+^. The Mo 3d spectrum showed two peaks at 231.6 and 234.75 eV, representing to the Mo 3d_5/2_ and Mo 3d_3/2_ orbitals. It was ascribed to the oxidation state of Mo^6+^. It was evident that O 1s exhibited a distinct peak at the binding energy of 529.1 eV, which was associated with the Bi-O bond in the [Bi_2_O_2_]^2+^ layered structural unit in Bi_2_MoO_6_. The two peaks of S 2p at 158.3 and 163.6 eV were both assigned to S 2p_3/2_ with spin-orbit splitting difference of 5.3 eV, these showed the S-doped in the photocatalytic were in the form of S^2−^. The two peaks of S 2p coincided with the spin-orbit bimodal of Bi 4f, indicated that S^2−^ were successfully doped in the lattice of Bi_2_MoO_6_ [34].

### 3.6. Brunauer Emmett Teller (BET) Specific Surface Area Analysis

**Figure 10:**
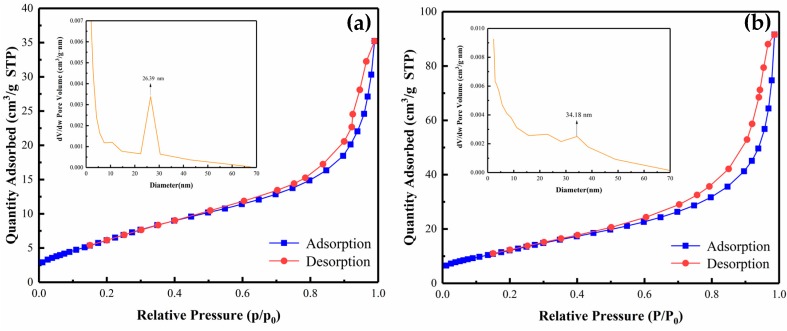
N_2_ adsorption–desorption isotherms and the corresponding pore size distribution curves (inset) of the Bi_2_MoO_6_ (**a**) and 1% S-doped Bi_2_MoO_6_ (**b**).

The typical N*_2_* adsorption–desorption isotherms and pore-size distribution curves of the pure Bi*_2_*MoO*_6_* and 1% S-doped Bi_2_MoO_6_ samples are shown in Figure 10. Both isotherms belong to the type IV curve, indicating the formation of mesoporous structures. The hysteresis loop shows type H3, which proves that the samples are aggregated by many nanosheets. This is consistent with the SEM results above. The porous structures provide sufficient transport pathways for photogenerated electrons and holes, which is helpful for photocatalytic degradation. The BET surface area of pure Bi_2_MoO_6_ was 26 m^2^/g. The 1% S-doped Bi_2_MoO_6_ exhibited a significant increase of the surface area (49 m^2^/g). The obtained BET specific surface areas for different samples are also shown in Table 1. Moreover, the insets in Figure 10 indicate that the pore size distribution of Bi_2_MoO_6_ and 1% S-doped Bi_2_MoO_6_ were respectively concentrated around 26.39 and 34.18 nm. It can be seen from Table 1 that the specific surface area and pore size of Bi_2_MoO_6_ increase regularly with the increase of S doping amount. A larger specific surface area and pore size may facilitate the progress of the photocatalytic reaction, but not the larger the specific surface area, the higher the photocatalytic activity. Combined with the photocatalytic test results above, it can be concluded that the physical adsorption performance is not the most important factor affecting photocatalytic degradation performance, and the other factors are required to synergistically affect photocatalytic performance.

### 3.7. Stability and Reuse Property

**Figure 11:**
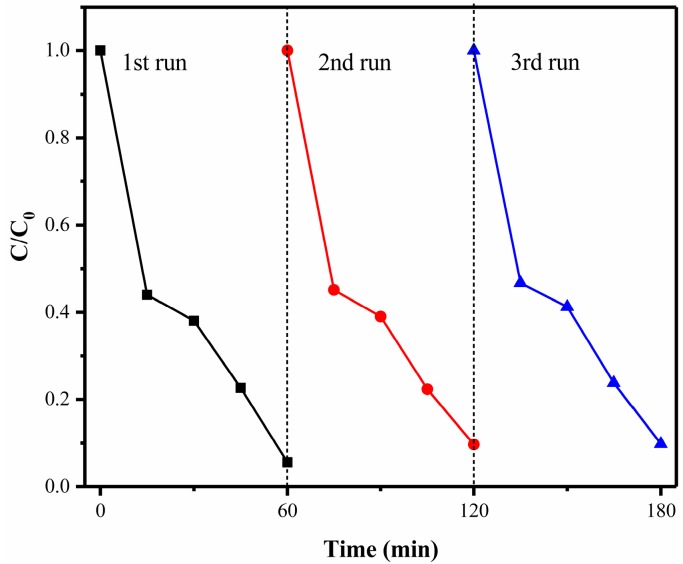
Cycling experiment of the degradation of Rhodamine B (RhB) with 1% S-doped Bi_2_MoO_6_ samples.

In order to study the reusability and stability of the 1% S-doped Bi_2_MoO_6_ sample, RhB was used as the target degradant, and the photocatalyst was repeatedly tested for photocatalytic performance under the same conditions. After each photocatalytic experiment was completed, the used catalyst was filtered, washed, dried, and then reused. It can be seen from Figure 11 that after one recycling, the degradation efficiency of the catalyst was reduced from 97% to 94%; after recycling twice, the activity of the catalyst was slightly lower; the degradation efficiency of the photocatalyst still remained at 90% after three degradation experiments. It did not show significant deactivation in the photocatalytic degradation reaction of three cycles, which indicated that the 1% S-doped Bi_2_MoO_6_ sample had excellent stability and recyclability [35,36]. 

**Figure 12:**
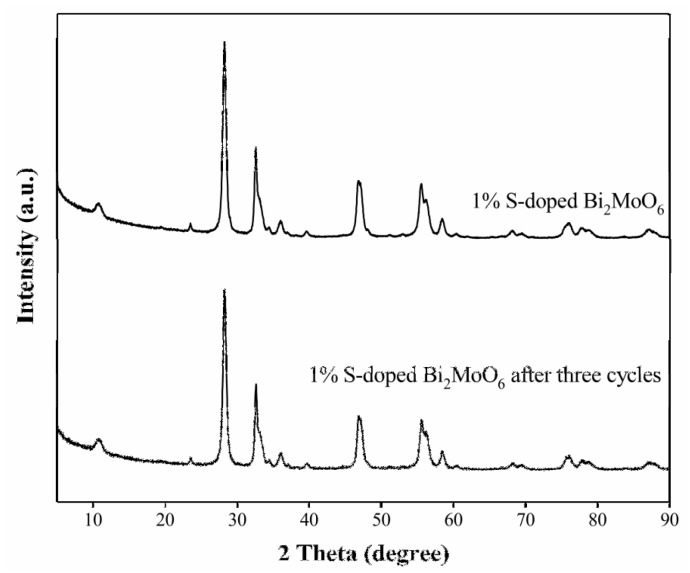
The XRD of 1% S-doped Bi_2_MoO_6_ fresh samples and the samples after three photocatalytic experiments.

The crystal composition and structure of the used photocatalyst was tested via XRD. It can be observed from Figure 12 that there is no difference in the phase and structure of the 1% S-doped Bi_2_MoO_6_ sample after three photocatalytic experiments and before participating in the reaction. Therefore, we think that 1% S-doped Bi_2_MoO_6_ has great stability and reusability [37,38].

### 3.8. Photoelectrochemical Property

**Figure 13:**
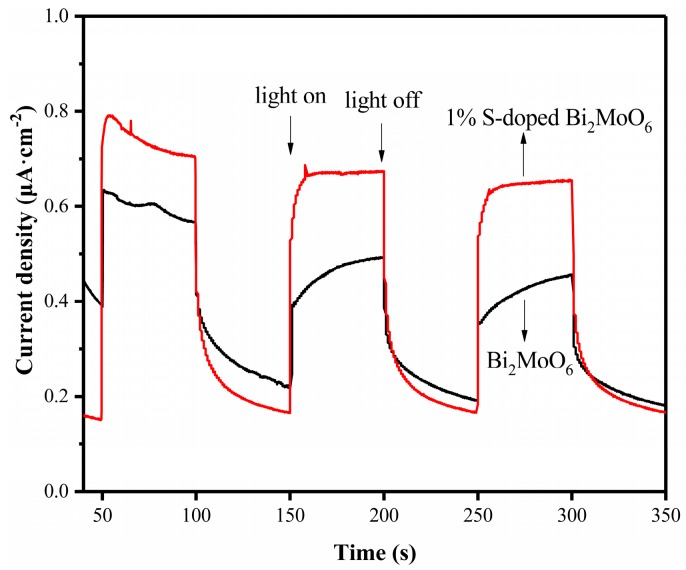
Photocurrent density of Bi_2_MoO_6_ and 1% S-doped Bi_2_MoO_6._

As we know that photocurrent response can confirm the generation and transfer of photogenerated charge carriers in photocatalytic reactions, which is directly related to photocatalytic activity [39,40]. Figure 13 shows the photocurrent responses of Bi_2_MoO_6_ and 1% S-doped Bi_2_MoO_6_ under light. It is obvious that the intensity of photocurrent response of 1% S-doped Bi_2_MoO_6_ (0.53 μA·cm^−2^) is significantly higher than that of the pure Bi_2_MoO_6_ (0.28 μA·cm^−2^), which demonstrated that S doping could greatly promote the separation and migration of photogenerated electrons and holes, and reduce the recombination rate of electron-hole pairs. The result was consistent with the PL analysis above [41,42].

**Figure 14:**
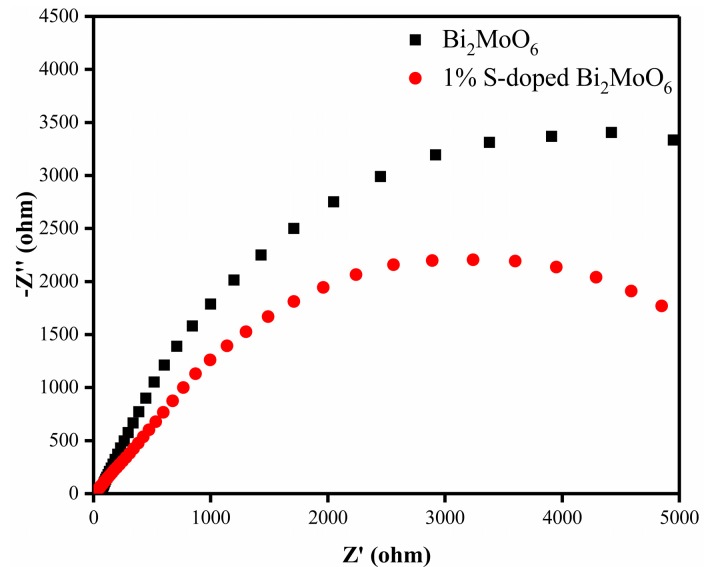
The Nyquist plots of Bi_2_MoO_6_ and 1% S-doped Bi_2_MoO_6._

Figure 14 shows the EIS (electrochemical impedance spectroscopy) curves, which were represented by real impedance (Z ′_real_) and imaginary (Z ′_image_) in the form of Nyquist plots. The curve radius reflects the electrical resistance of the interface layer on the electrode surface. In general, the smaller curve radius means a smaller resistance of the sample surface and a smaller transfer impedance of electrons, indicating an effective separation of photogenerated electron-hole pairs and a faster transfer rate of charge carriers [43,44,45]. As we can see from Figure 14, the curvature radius of 1% S-doped Bi_2_MoO_6_ is significantly less than that of the pure Bi_2_MoO_6_, proving the 1% S-doped Bi_2_MoO_6_ sample has stronger photogenerated charge transfer capability. Meanwhile, it was found that the charge transfer resistance (Rct) of Bi_2_MoO_6_ decreased from 7388 Ω (pure Bi_2_MoO_6_) to 5785 Ω (1% S-doped Bi_2_MoO_6_) after S doping, indicating that the charge separation efficiency was remarkably increased. This is beneficial to increase photocatalytic activity.

In summary, it is confirmed by photocurrent intensity and electrochemical impedance spectroscopy that 1% S-doped Bi_2_MoO_6_ has higher separation and migration abilities of electron-hole pairs compare to pure Bi_2_MoO_6_. This conclusion is completely consistent with the previous photocatalytic test result that 1% S-doped Bi_2_MoO_6_ has higher photocatalytic performance. 

### 3.9. Photocatalytic Mechanism

**Figure 15:**
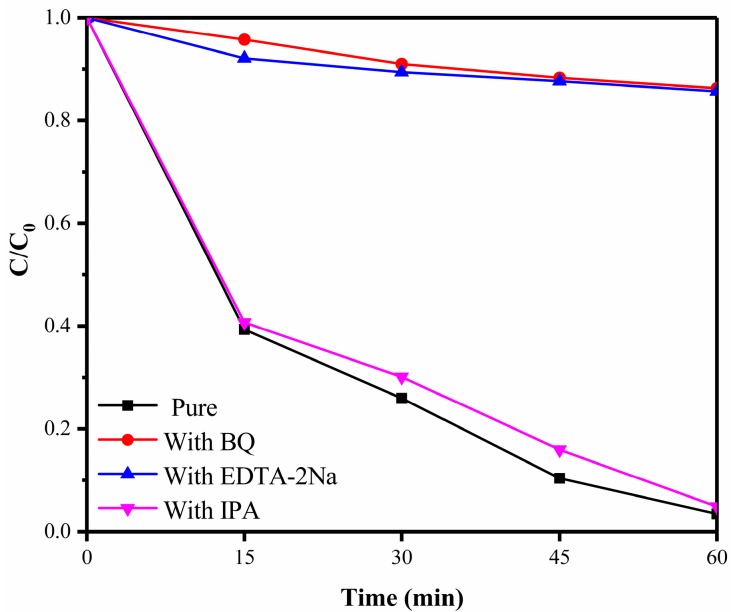
Active species capture experiments of 1% S-doped Bi_2_MoO_6_ sample.

For the purpose of exploring the detailed mechanism of photocatalytic degradation of RhB with 1% S-doped Bi_2_MoO_6_ samples, the capture experiments were conducted to verify the dominated active substances involved in photocatalytic reaction, such as holes (h^+^), superoxide radicals (·O_2_^−^) and hydroxyl radicals (·OH). After adding the different scavengers, the RhB degradation rates by 1% S-doped Bi_2_MoO_6_ samples presented different trends and are shown in Figure 15. The photocatalytic degradation rate of RhB decreased significantly to only 14% after 1 h reaction with the introduction of the superoxide radical (·O_2_^−^) scavenger (benzoquinone), indicating that ·O_2_^−^ played a leading role in the photocatalytic reaction. After adding the hole (h^+^) scavenger (EDTA-2Na), the photo-degradation rate of RhB was apparently restrained to only 15%, which suggested that holes (h^+^) were one of the dominant active substances in the photocatalytic process of 1% S-doped Bi_2_MoO_6_. However, the addition of isopropanol, the scavenger of hydroxyl radical (·OH) had little effect on the degradation efficiency of RhB. Therefore, we can draw conclusion that superoxide radicals ·O_2_^−^ and holes h^+^ are the dominant active substances in the process of degrading pollutants by 1% S-doped Bi_2_MoO_6_ samples.

**Figure 16:**
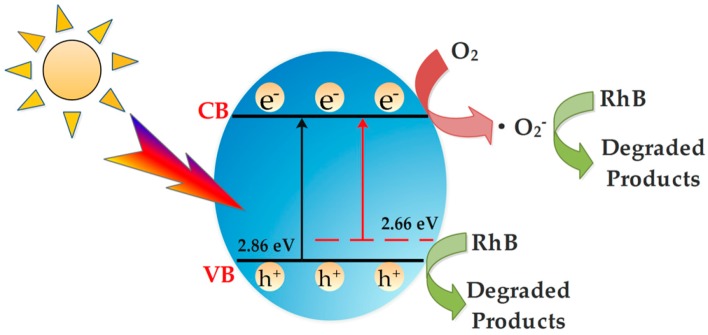
Proposed photocatalytic mechanism of S-doped Bi_2_MoO_6_ sample.

It can be seen from Figure 16 that the S^2−^ doped into the lattice of Bi_2_MoO_6_ replaced the O^2−^, which caused expansion of lattice and changed the original electronic band structure of Bi_2_MoO_6_. Meanwhile, S doping caused the increase of oxygen vacancies in the lattice gap, so that S-doped Bi_2_MoO_6_ has better absorption of visible light. In addition, the sulfur in the lattice gap of Bi_2_MoO_6_ generated local points at the edge of the valence band. Under the synergy with oxygen vacancies, a new S 2p energy band was formed on the original O 2p valence band, the band gap width of Bi_2_MoO_6_ was narrowed, and the band gap energy of the electronic transition was dropped from 2.76 to 2.66 eV, which decreased the energy required for electronic transitions. After absorbing the same intensity of light, S-doped Bi_2_MoO_6_ generated more photogenerated electrons and holes, which increased the active radicals, thereby the photocatalytic activity was increased.

## 4. Conclusions

S-doped Bi_2_MoO_6_ photocatalysts were successfully prepared via a simple hydrothermal process. The morphology, structure, and properties of the samples were investigated via XRD, Raman, FT-IR, XPS, SEM, TEM, N_2_ adsorption–desorption, PL, and UV-vis DRS analyses. The following conclusions were obtained: (1) 1% S-doped Bi_2_MoO_6_ exhibited the excellent photocatalytic activity under visible light irradiation. It was ascribed to the fact that the S^2−^ doped into the lattice of Bi_2_MoO_6_ replaced the O^2−^, which caused expansion of lattice and reduction of band gap energy. Meanwhile, the recombination of photogenerated electrons and holes were inhibited after sulfur doping, thereby the photocatalytic performance was improved. (2) The recycling experiments indicated that the photo-degradation rate of RhB by 1% S-doped Bi_2_MoO_6_ after three reuses can still reached 90%, indicating that it had great reusability and stability. In addition, the superoxide radicals (·O_2_^−^) and holes (h^+^) were verified to be the leading active substances in the photocatalytic process of 1% S-doped Bi_2_MoO_6_.

The 1% S-doped Bi_2_MoO_6_ is also superior over Bi_2_MoO_6_ doped with other elements or hybrids reported in the previous literatures [11,12,13,14,15,16,17,18]. In this study, it is not necessary to load a precious metal, but only a low-cost thiourea is required to prepare a photocatalyst possessing high catalytic activity. Meanwhile, compared with the methods in other literatures, this study required a short time, low economical energy consumption, and easy operation in the process of preparing samples. In addition, under the same photocatalytic reaction conditions, the degradation rate of RhB (100 mL 10 mg/L) by 1% S-doped Bi_2_MoO_6_ (0.1 g) can reach 97% after 60 min irradiation. Doping other substances, such as carbonate-doped Bi_2_MoO_6_, can completely degrade RhB (0.02 mol/L) after 90 min of irradiation with precious metals (Pd, Ag, Au) [13]; the degradation rate of RhB (5 mg/L) by Ti^4+^ and Ni^2+^ co-doped Bi_2_MoO_6_ can reach 98.8% after 60 min of light [16]. It can be seen that compared to Bi_2_MoO_6_ doped with other elements or hybrids, 1% S-doped Bi_2_MoO_6_ always exhibits higher photocatalytic activity. In summary, S-doped Bi_2_MoO_6_ has higher economic benefits and better application prospects.

## Figures and Tables

**Table 1:** The average crystallite size, band gap energy, and specific surface area of Bi_2_MoO_6_ with different S doping amount.

Sulfur Doping Amount	0	0.5%	1%	2%	5%
Average grain size (nm)	28.2	17.7	16.1	10.8	9.2
Band gap energy (eV)	2.86	2.75	2.66	2.60	2.49
Specific surface area (m^2^/g)	26	31	49	50	54
